# *MET* Genetic Abnormalities Unreliable for Patient Selection for Therapeutic Intervention in Oropharyngeal Squamous Cell Carcinoma

**DOI:** 10.1371/journal.pone.0084319

**Published:** 2014-01-17

**Authors:** Ludovic Lacroix, Sophie F. Post, Alexander Valent, Antoine E. Melkane, Philippe Vielh, Coumaran Egile, Christelle Castell, Christelle Larois, Sandrine Micallef, Patrick Saulnier, Hélène Goulaouic, Anne-Marie Lefebvre, Stéphane Temam

**Affiliations:** 1 Department of Medical Biology and Pathology, Institut Gustave Roussy, Villejuif, France; 2 Translational Research Laboratory and Biobank, Institut Gustave Roussy, Villejuif, France; 3 Department of Head and Neck Surgical Oncology, Institut Gustave Roussy, Villejuif, France; 4 Department of Plastic Surgery, University Medical Centre Groningen, Groningen, The Netherlands; 5 Department of Oncology, Sanofi, Vitry-sur-Seine, France; 6 Department of Biologics Scientific Core Platform (BioSCP), Sanofi, Vitry-sur-Seine, France; 7 Department of Scientific Core Platform Clinical and Scientific Operations (SCP CSO), Sanofi, Vitry-sur-Seine, France; Shanghai Jiao Tong University School of Medicine, China

## Abstract

**Background:**

Identification of *MET* genetic alteration, mutation, or amplification in oropharyngeal squamous cell carcinoma (OPSCC) could lead to development of *MET* selective kinase inhibitors. The aim of this study was to assess the frequency and prognostic value of MET gene mutation, amplification, and protein expression in primary OPSCC.

**Methods:**

A retrospective chart review was conducted of patients treated for single primary OPSCC between January 2007 and December 2009. Pre-treatment OPSCC tissue samples were analyzed for MET mutations, gene amplification, and overexpression using Sanger sequencing, FISH analysis, and immunohistochemistry respectively. Univariate and multivariate analyses were used to analyze correlations between molecular abnormalities and patient survival.

**Results:**

143 patients were included in this study. Six cases (4%) were identified that had a genetic variation, but previously described mutations such as p.Tyr1235Asp (Y1235D) or p.Tyr1230Cys (Y1230C) were not detected. There were 15 high polysomy cases, and only 3 cases met the criteria for true MET amplification, with ≥10% amplified cells per case. Immunohistochemistry evaluation showed 43% of cases were c-MET negative and in 57% c-MET was observed at the tumor cell level. Multivariate analysis showed no significant association between MET mutation, amplification, or expression and survival.

**Conclusions:**

Our study shows a low frequency of MET mutations and amplification in this cohort of OPSCC. There was no significant correlation between MET mutations, amplification, or expression and patient survival. These results suggest that patient selection based on these MET genetic abnormalities may not be a reliable strategy for therapeutic intervention in OPSCC.

## Introduction

Head and neck cancer (HNC) is the sixth most common cancer in the world, with approximately 635,000 new cases worldwide in 2008.[1] Oropharyngeal cancer (OPC) was responsible for an estimated 36,540 new cases and 7,880 deaths in 2010 in the United States[2], and 91,900 new cases and 41,700 deaths in 2008 in Europe[3]. The incidence is higher in males (3.9% of total cancers) than females (1.6% of total cancers).[3] OPC includes tumors arising within the posterior pharyngeal wall, soft palate, tonsillar region, and the base of the tongue. The majority of these tumors (>95%) are squamous cell carcinomas (SCC) of mucosal origin.[4] A rise in the incidence of OPC has been observed in the developed world, particularly in North America, which may be explained by oncogenic Human Papilloma Virus (HPV) infection. HPV-positive and HPV-negative OPC's represent distinct subgroups. The first has a better prognosis, whereas the second is associated with tobacco and alcohol abuse and has a poorer prognosis.[5]

*MET* is a receptor tyrosine kinase located on chromosome 7q31 that encodes several functional domains: the semaphoring (SEMA) domain (ligand-binding), juxtamembrane (JM) domain (regulatory), and the tyrosine kinase (TK) domain.[6], [7] The only known ligand for *MET* is the hepatocyte growth factor/scatter factor (HGF/SF).[8] *HGF/MET* signalling leads to various cellular responses, such as increased cell growth, cell motility, survival, angiogenesis, wound healing, tissue regeneration, and invasion/metastasis.[6], [7] *MET* activation can occur via multiple molecular events including: gene mutation, gene amplification, protein overexpression, or ligand dependent autocrine and paracrine loop.[9]–[12]

*MET* is overexpressed in various solid tumors (eg. small cell lung cancer, non-small cell lung cancer, gastric cancer, renal cell carcinoma, and breast cancer)[6], [13]–[17] and its overexpression is often associated with an aggressive phenotype and poor prognosis. In head and neck squamous cell carcinoma (HNSCC) specifically, several reports indicate 70% to 100% *MET* overexpression, suggesting that the *HGF/MET* pathway plays a role in carcinogenesis.[8], [12], [18]–[21] *HGF/MET* signalling could also interfere with response of oropharyngeal squamous cell carcinoma (OPSCC) to radiotherapy (RT).[22]

Furthermore, *MET* genetic alterations have been previously reported in HNC. *MET* Y1235D mutation (also known as Y1253D) was found in 15 out of 138 patients (11%) with OPSCC[23], and in 21 out of 152 patients (14%) with HNSCC[24], suggesting a relatively high prevalence of this mutant in the OPC. This *MET* Y1235D mutation and another mutation in the *MET* activation loop (Y1230C) were also detected in neck lymph nodes metastases from a primary HNSCC tumor.[25] Recently, another epidemiological study reported novel mutations in the SEMA, JM, and TK domains of *MET* in 13.5% of the cases, and an increased *MET* gene copy number (>10 copies) in 3 out of 23 cases.[8]

The identification of a cancer type in which *MET* genetic alteration, mutation, or amplification is present in a significant subset such as OPSCC is of high interest. This could lead to development of *MET* selective kinase inhibitors, since genetic alterations are often responsible for oncogenic addiction. There are various recent clinical trials that have already demonstrated the effect of *MET* inhibitors in patients with a variety of advanced or metastatic tumors, including non-small-cell lung cancer, and breast, prostate, liver, and renal cancer.[10] The aim of this study is to determine the frequency and prognostic value of *MET* gene mutation, amplification, and protein expression in OPSCC.

## Materials and Methods

### Ethics Statement

The Gustave Roussy Cancer Institute (IGR) Institutional Review Board gave approval for this study. Written informed consent was obtained from included patients, and patient confidentiality was protected throughout the study.

### Patient selection

A retrospective chart review was conducted for patients treated for a single primary OPSCC between January 2007 and December 2009 at the IGR. Patients with recurrences, multiple, synchronous, or metachronous lesions were excluded from this study.

Pretreatment tissue samples were retrieved from the IGR Biobank. Most tissue samples were obtained by surgical biopsy and fixed using AFA, while tissue samples obtained during surgical resection were fixed with 10% formalin. Based on hematoxylin and eosin (H&E) staining, tissue samples with at least 70% tumor cells were selected. DNA sequencing, fluorescence in situ hybridization (FISH) assay, immunohistochemistry (IHC), and HPV detection were done in different laboratories, in a strictly blind fashion.

### DNA extraction and sequencing

Genomic DNA was extracted from 10 to 20 µm tissue bloc sections from tumors using the DNeasy Blood and tissue kit (QIAGEN, Hilden, Germany) according to the manufacturer's instructions. The full coding sequences of *MET* exons 2 and 13 through 21 (NM_000245.2), exons already described as known locations for oncogenic mutations, were analyzed. Sanger sequencing was done following polymerase chain reaction (PCR) amplification of target exons. The primer sequences are reported in Table 1. The sequencing reactions were carried out using the BigDye Terminator Cycle Sequencing Kit as indicated by the manufacturer (Applied Biosystems, Forster City, CA). Sequencing reactions were analyzed on a 48-capillary 3730 DNA Analyzer. The sequence alignment and analysis were performed with SeqScape software (Applied Biosystems). All detected mutations were confirmed at least once with independent PCR amplification.

**Table 1:** Primer sequences for *MET* gene* (reference sequence NM 001127500.1, gene ID 4233, Chr7q31).

Primer Couple ID	Exon Targeted	Foward Primer	Foward Primer Lengh	Reverse Primer	Reverse Primer Lenght
S327	2	AATGGTATAGGTCTTTCAGTTTTCTCTTC	29	TGTAGAATGACATTCTGGATGGGT	24
S328	2	AGTCCGAGATGAATGTGAATATGAAG	26	AGCCATGTTGATGTTATCTTTCCA	24
S150	2	GATTGTTTCCCATGTCAGGACTG	23	TGGTATTGCCTACAAAGAAGTTGATG	26
S151	2	CTGTGTGGTGAGCGCCC	17	AACCTGATTATTCTTGTGTGAAAAGTCT	28
S152	2	CGGTCCAAAGGGAAACTCTAGAT	23	ACATATTTGATAGGGAATGCACACAT	26
S153	2	TAGGAGCCAGCCTGAATGATG	21	CTGCAACTATTTTGGATAAACACCAT	26
S329	13	CAACCTGTGTAGTACAAATATCTATCATGG	30	GACAATCTTAAACTGTAATGACTGTGTTCTTA	32
S022	14	CACTGGGTCAAAGTCTCCTGG	21	TGTCACAACCCACTGAGGTATATGT	25
S330	15	TTTCAGTCCCCATTAAATGAGGTTT	25	GGCCAAAGATAAAATGCTTACTGGA	25
S023	16	AAAATGAAGCTCATAAAGGGTTTGA	25	GGCCCATAATTTCAGTGGTAGC	22
S154	17	CTCTTCCTATCTAAATTTGACAAAAGTATTCA	32	GAAGGGATGGCTGGCTTACA	20
S155	18	CTTGAGCCATTAAGACCAAACTAATTT	27	ACAGTGGGAAACAGATTCCTCC	22
S024	19	AATTATTCTATTTCAGCCACGGGT	24	AAAACTGGAATTGGTGGTGTTGA	23
S331	20	TTAGTTACCAAGACCTACTGATTTCCTTTC	30	TTTGAAGGCAGGCATTTCTGTA	22
S332	21	TTTACAGAAATGCCTGCCTTCAA	23	TCAGGCAGTGAAAAAACCATTG	22

*MET proto-oncogene (hepatocyte growth factor receptor); other aliases: AUTS9, HGFR, RCCP2, c-MET

### Detection of HPV DNA viral load using RealTime qPCR

Tests were done to detect the three oncogenic HPV strains (16, 18, and 33) as well as the reference gene gACTB (β-actin) as previously described by Melkane et al.[26] Briefly, quantitative HPV DNA viral load measures were available, and a semi-quantitative viral load categorization was obtained according to individual Cycle Thresholds (CT); CT≥45: −, CT<45: +. All mildly HPV-positive samples (38≤CT<45) were confirmed on independent testing.

### MET gene FISH assay

FISH for the *MET* gene was performed on 4 µm tumor sections, using the ZytoLight SPEC MET/CEN 7 Dual Color Probe (ZytoVision GmbH, Bremerhaven, Germany) according to the manufacturer's instructions. SNU-5 (ATCC® CRL-5973™) cell line xenografts bearing MET amplification were used as positive control samples to set up the FISH staining protocol. Briefly, the probe was co-denatured for five minutes at 84°C on the slide, and then incubated overnight at 44°C. Slides were washed with post-hybridization wash buffer (0.5X SSC/0.1% SDS) for five minutes at 37°C, after which they were air-dried and counterstained with DAPI dissolved in an anti-fade mounting solution. At least 100 tumor cells were analyzed, and the number of fluorescent signals within the nuclear boundary of each evaluable interphase tumor cell was counted using an Axiophot-ZEISS fluorescent microscope at 1000× magnification. Only nuclei with unambiguous centromeric (D7Z1 locus) hybridization red signals (as control) and *MET* (7q31) probe green signals were scored. Tumors were classified as previously described by Capuzzo et al.[27] Briefly, FISH negative was when there were two or three copies of the *MET* gene present in major clone of tumor cells, or when four *MET* gene copies were present in less than 40% of tumor cells. The term ‘high polysomy’ was applied if four to six copies of the *MET* gene were present in more than 40% of tumor cells. In order to declare ‘gain of gene’, more than one or two supplementary copies of the *MET* gene (compared to the centromeric probe) had to be present in tumor cells. Amplification was defined as a *MET* gene to centromere ratio >2.

### IHC

#### Immunostaining

We performed *MET* and p-MET immunostaining using serial tissue sections. Briefly, antigen retrieval was performed with Cell Conditioning 1 (CC1) buffer at 95°C for 8 minutes, and then at 100°C for 28 minutes. After the endogen biotins blocking step, slides were incubated with the primary anti-antibody for one hour at 24°C for the rabbit anti-human c-MET (final dilution 1/50, clone SP44, reference M3444, Spring Bioscience, USA or clone CVD13, reference 18-2257, Invitrogen, USA) and at 37°C for anti-p-MET (Tyr1234/1235) (final dilution 1/50, clone D26, reference 3077, Cell Signaling Technologies, USA). A post-fixation step with glutaraldehyde (0.05% in NaCl 0.9% w/v) for 4 minutes at 24°C was done. For *MET* detection, the secondary antibody biotin-SP-conjugated Affinipure goat anti-rabbit (reference 111-065-003, batch 84328, Jackson ImmunoResearch Laboratories, Inc, USA) was incubated at 24°C for 32 minutes at 0.5 microg/mL. For p-MET, the secondary antibody biotin free peroxidase multimer anti-rabbit UltraMap™ was incubated at 24°C for 16 minutes. Immunostaining was done with 3,3-diaminobenzidine tetrahydrochloride (DAB) from DABMap™ chromogenic detection kit according to manufacturer's recommendations. A counter-staining step was done with hematoxylin and blueing reagent was applied. Stained slides were dehydrated and coverslipped with cytoseal XYL (8312-4, Richard-Allan Scientific, USA).

#### Xenograft tumors

Investigation using xenograft tumors was done to determine the impact of fixative type on p-MET detection. SNU-5 (ATCC® CRL-5973™) tumor cell line xenografts were divided into two groups, and placed in neutral buffered formalin (HT50112, Sigma-Aldrich, France) or AFA (acetic acid, formaldehyde, alcohol) fixative.

#### Image analysis

Immunostained slides were scanned using the ScanScope XT system (Aperio Technologies, Vista, CA). Digitized images were captured using the ImageScope software (version 10.2.2.2319, Aperio Technologies) at 20× magnification.

#### IHC scoring

Staining evaluation included the histological site of reactivity, main type of reactive cell, staining, intensity, and cell staining frequency. The negative samples were scored as 0+. The positive samples were scored with a scale of intensity from 1+ to 3+. Ranges of intensities were described as weak (0 to 1+), moderate (1+ to 2) and strong (2+ to 3+). Cell frequency was the percentage of immunostained cells, and was estimated by observation by a histologist as the median per sample. The cell frequency was divided into five categories of proportion score: 1 (0–5%), 2 (6–25%), 3 (26–50%), 4 (51–75%) and 5 (76–100%).

### Statistical analyses

Descriptive statistics were used to summarize the study cohort. The Kaplan-Meier method was used to estimate overall survival (OS), progression free survival (PFS) and specific survival (SS). The evaluation and selection of covariates to be included in the multivariate model for survival was done as follows. The first step was to assess the correlation between covariates, in order to avoid keeping in the model two covariates when they are highly correlated and bring similar information. A threshold of 0.4 was used for this selection. Then, univariate analyses were conducted using Cox Proportional Hazards Model on the above factors to identify the variables with the highest correlation with OS, PFS, and SS. Lastly, the multivariate Cox Proportional Hazards Model was run using the stepwise procedure with a variable entry of 5% level and a variable removal at each step of 10% level. When two covariates were highly correlated according to the threshold previously defined, only the most significant one in univariate analysis was kept for the multivariate analysis.

## Results

### Patient and tumor characteristics

Medical files of 150 consecutive patients with OPSCC were reviewed. Of these patients, three presented with synchronous tumor lesions, three had recurrent disease, and one patient had insufficient tissue material available for laboratory studies, and were excluded. There were 143 patients included in this study (Table 2). The majority of patients were male (69%), and the overall mean age of presentation was 59 years (range 27 to 87 years). Most patients had previous tobacco exposure, with 47/143 active smokers and 48/143 former smokers (quit for ≥6 months). Of the patients with previous alcohol use, 42/143 were active consumers and 18/143 were former consumers (quit for ≥6 months). Fifty-nine patients (41%) experienced combined alcohol and tobacco exposure (either active or past), whereas 47 patients (32%) had never been exposed to either carcinogen. Seventy tumors (79%) display p16 staining. Nevertheless, because we have previously shown that HPV DNA viral load status had a higher prognostic value than the p16 expression status, we focus on HPV DNA status for analysis.[26] Eighty-eight tumors (62%) were detected with HPV DNA on qPCR, irrespective of the viral load level, the involved HPV serotype, or the exclusive or combined infection status. There were 55 (38%) cases that were HPV negative.

**Table 2:** Patient and tumor characteristics.

Parameter	n (%)		
Total	143 (100)		
Sex			
Male	98 (69)		
Female	45 (31)		
Age			
<50	21 (15)		
≥50	122 (85)		
Tobacco exposure			
Yes	95 (66)		
No	48 (34)		
Alcohol exposure			
Yes	60 (42)		
No	83 (58)		
Tumor location			
Tonsillar fossae and pillars		90 (63)	
Base of tongue		21 (14)	
Glosso-tonsillar sulcus		11 (8)	
Valleculae		8 (6)	
Soft palate		7 (5)	
Posterior pharyngeal wall		6 (4)	
Differentiation			
Well		70 (49)	
Moderate		50 (35)	
Poor		23 (16)	
T stage			
T1/T2		59 (41)	
T3/T4		84 (59)	
N stage			
N0		48 (34)	
N1-N3		95 (66)	
AJCC* classification			
Stage I/II		29 (20)	
Stage III/IV		114 (80)	

*American Joint Committee on Cancer

The majority of tumors were localized in the tonsillar fossae and pillars (63%), or at the base of the tongue (14%). The macroscopic aspect of the tumors was exophytic in 88 cases (62%), and ulcerative or infiltrative in 55 cases (38%). Histopathologically, approximately half of the tumors was well-differentiated, and the other half was moderately or poorly differentiated. The majority of patients presented with T3 or T4 stage tumors, and had N1-N3 lymph node metastases. There were six patients (4%) with distant metastases at the time of evaluation, all with pulmonary metastases. According to the 2010 AJCC classification system, 29/143 patients (20%) presented with early-stage (stage I and II) tumors, while a majority of 114/143 patients (80%) had advanced stage (stage III and IV) disease.

Most of the patients (136/143, 95%) in this study were treated in a curative setting. Patients were treated by RT; either exclusively (31/143 patients, 21%), or by concomitant chemoradiotherapy (CRT) (75/143 patients, 52%), and surgery; either exclusively (15/143 patients, 11%), or followed by adjuvant RT or CRT (16/143 patients, 11%). The remaining six patients (5%) with distant pulmonary metastases were offered treatment with palliative chemotherapy.

### *MET* mutations in OPSCC

Exons 2, 13, 14, 15, 16, 17, 18, 19, 20, and 21 were analyzed in all 143 cases, but due to low tissue sample quality, some cases had several failed PCR's (>5 failures) and were removed from further analyses. Overall, six cases (4%) were identified that had a genetic variation (Table 3).

**Table 3:** The location and predicted effect of each *MET* variation found, numbered from the reference sequence NM 000245.2.

Variation	# of cases	Location	Align-GVGD† [48]	SIFT‡ [49]	PolyPhen-2[50]
p.Val136Ile	1	Semaphoring domain	Class C0 (benign/no effect)	tolerated	benign/no effect
p.Glu312Lys	1	Semaphoring domain	Class C0 (benign/no effect)	tolerated	benign/no effect
p.Thr1036Ile	1	Juxtamembrane domain	Class C15 (damaging)	affects protein function	benign no effect
p.Cys1210Arg	1	Kinase domain	Class C0 (benign/no effect)	affects protein function	probably damaging
p.Ala347Thr	2	Semaphoring domain	Class C55 (damaging)	affects protein function	probably damaging

Mutation Chromatograms include reference sequences and variant description sequence variations described using IUPAC code (http://www.insdc.org/).

Align-Grantham Variation Grantham Deviation.

Sorts Intolerant From Tolerant.

Four of these mutations were located on exon 2, one on exon 15, and one on exon 18. The p.Val136Ile mutation has been previously detected once before in a kidney carcinoma case[6], and the p.Ala347Thr mutation has been described in lung squamous cell carcinoma[28]. The p.Glu312Lys, p.Thr1036Ile, and p.Cys1210Arg variations have not yet been described in literature and are novel somatic variant with unknown pathogenicity.[29] Previously described mutations such as p.Tyr1235Asp (Y1235D) or p.Tyr1230Cys (Y1230C) in exon 19 were not found. Of the six variations, four were expected to affect protein function based on the SIFT classification system, three of which had predicted damaging effects according to the PolyPhen-2 scoring system.

### *MET* amplification in OPSCC

**Figure 1:**
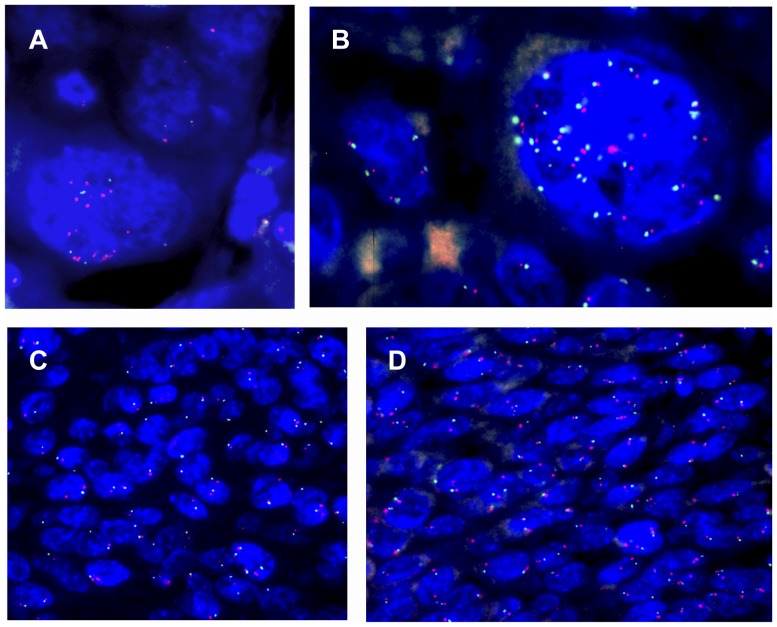
FISH with two color probes: chromosome 7 centromere (green) and *MET* gene (red). A) True *MET* gene amplification in 10% of cells: 4–8 centromere signals and 16–20 *MET* signals, ratio >2.0. B) High polysomy: the same number of control and *MET* gene spots were seen in 15% of giant cells, ratio is 1.0. C) Chromosome 7 monosomy: only one control and one *MET* signal were detected for this case. In some cells there is only one signal (or no signal) due to a nuclei section. D) Normal hybridization pattern: two control spots and two *MET* gene spots. Again, some cells show only one of two signals due to a nuclei section.

FISH analysis was performed for 128 out of 143 cases, because there was insufficient tissue from the remaining cases for further analysis. Hybridization was successful for 97 out of 128 cases. Of those cases, 39 were considered normal, 17 had monosomy of chromosome 7 or deletion of *MET* gene, 23 had low polysomy, and 15 had high polysomy of chromosome 7 (Table 4, Figure 1). In the high polysomy category, two cases were detected as gene amplifications, but the amplified clone presented only 4–5% of cells and thus did not meet the criteria to be included in the “amplified” category. Only three cases met the criteria for true *MET* amplification, with ≥10% amplified cells per case.

**Table 4:** Fluorescence in situ hybridization results.

Gene status	# of cases	Classification
Gene amplification	3	+
High polysomy	15	+
Normal	39	−
Monosomy/deletion	17	−
Low polysomy	23	−
Total	97	

### *MET* protein expression in OPSCC

**Figure 2:**
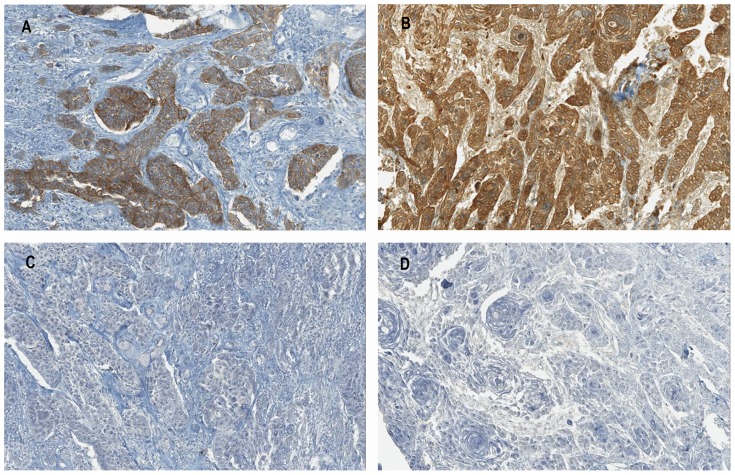
IHC staining for c-MET and pYY1234-1235 *MET* in OPSCC specimens. Moderate (A) and strong (B) membranous and cytoplasm c-MET immunostaining in tumor cells. No p-MET immunostaining was observed in serial section (C and D). (original magnification ×20).

IHC evaluation was done for 113 cases, and a total of 107 cases passed the quality controls and were included (Table 5). Forty six cases (43%) were c-MET negative. c-MET protein was observed in 61 cases (57%) at the tumor cell level. Of the 61 c-MET positive cases, immunostaining was localized at the membrane ± cytoplasm level in 50 cases (82%), with moderate expression (median intensity 1+ to 2+, and median cell frequency 50% to 75%). In the other 11 cases (18%), immunostaining was observed only at the cytoplasm level, with moderate expression (median intensity 1+, and median cell frequency 75% to 100%; Figure 2). Detection of pYY1234-1235 *MET* was observed in two cases (3%), but in non-tumor cells (at margin of tissue sample). AFA fixation resulted in complete disappearance of p-MET immunostaining (Figure 2). p-MET analysis was therefore only done for formol-fixed tissue.

**Table 5:** Immunohistochemistry results[26].

Parameter	n (%)
Analyzable cases	107 (100)
c-MET negative cases	46 (43)
c-MET positive cases	61 (57)
Membrane with or without cytoplasm expression	50 (82)
Cytoplasm expression	11 (18)
p-MET positive cases	2 (3)

### Survival analysis

The median follow-up time was 2.6 years (min. 11 days and max. 13.9 years), and there were 57/143 (40%) deaths observed. The median OS was 5.3 years. The probabilities of surviving (95% confidence interval) at one, two, and three years were 82.4% (CI: 76.1–88.7), 67.3% (CI: 59.5–75.1) and 64.5% (56.5–72.6) respectively. There were 71 oncological events (tumor progression, loco-regional or distant recurrence, and death) observed in the 143 patients. The median PFS was 4.4 years. The probabilities of having an oncological event (95% confidence interval) at one, two, and three years were 68.3% (CI: 60.7–76.0), 61.8% (CI: 53.8–69.9), and 57.5% (CI: 49.2–65.8) respectively. There were 30/143 (21%) cancer-related deaths. The positive HPV status, alcohol exposure, high tumor differentiation, T3/T4 staging, N1-3 staging, presence of metastases and advanced disease (AJCC classification III/IV) were significant at 5% for OS in the univariate Cox Proportional Hazards Model. Except N1-3 staging, these variables were also significant for PFS (at 5%). Univariate analysis did not show any significant association between OS, PFS, or SS and *MET* mutations, *MET* amplification, or *MET* overexpression. It is notable that *MET* positive cases were not specifically distributed in either the HPV positive or HPV negative groups. The multivariate analysis did not show any significant association between OS, PFS, or SS and *MET* mutations, *MET* amplification, or *MET* overexpression.

## Discussion

The aim of this study was to determine the frequency and prognostic value of *MET* abnormalities including DNA mutations, gene amplification, and protein expression in primary OPSCC. Our study shows a low frequency of *MET* mutations and *MET* amplification in OPSCC. There was no significant correlation between *MET* mutations, amplification, or expression and patient survival.

In our study six cases were identified with *MET* genetic variations, four of which have not been previously described, though the clinical relevance of these variations remains unclear. Unfortunately, non-tumor tissue was not available to confirm if those mutations were somatic. Overall, our *MET* mutation incidence (4%) was low compared to a number of other studies. For example, Seiwert et al. found a 13.5% mutation incidence in the semaphoring, juxtamembrane, and tyrosine kinase domains of the *MET* gene in 66 HNSCC tumor tissues, as well as *MET* over expression in 84% of samples.

Previously described HNSCC mutations such as p.Tyr1235Asp (Y1235D)[23]–[25] and p.Tyr1230Cys (Y1230C)[25] were not detected in our study. In a study investigating the prevalence and clinical impact of the Y1253D mutation in patients with OPSCC treated by radical RT, the mutation was detected in 15/138 tumors (10.9%). Also, survival analysis showed a significant correlation between Y1253D mutation and impaired local tumor control.[23] Another study by Ghadjar et al. detected Y1253D mutation in 21 tumors in 152 patients (14%) with HNSCC and observed an association with decreased distant metastasis-free survival.[24] On the other hand, a set of 12 oral SCC's were examined for mutation and while overexpression of the *MET* receptor was present in all cases, point mutations were not detected.[20]

Studies investigating *MET* mutations use different DNA detection techniques. Our Sanger sequencing approach is known to detect mutation from 20–30% of mutated DNA. Our cohort included only samples with more than 70% of tumor cells. Nevertheless, our Sanger approach does not detect minor clones representing less than 20% of the tumor cell, but can detect unexpected mutations. More sensitive techniques such as the technique used by Aebersold et al.[23] can detect minor clones, but can only detect mutations that are targeted specifically. We could hypothesize that the technique used in our study may have yielded a relatively lower DNA mutation frequency than a more sensitive technique, if *MET* mutations are mainly present in minor clones. One the other hand, we could also stipulate that the present French cohort had a lower rate of *MET* mutation that other published cohorts.

We also found a low frequency of amplification, with no correlation with patient survival. Out of 97 cases, there were only three cases that met the criteria for true *MET* amplification and 15 cases with high polysomy. A previous study found >10 *MET* gene copies in 3 of 23 (13%) HNSCC tumor tissues.[8] In other studies, *MET* gene amplification has been associated with poor prognosis in gastric and lung cancer. Lee et al. recently found 61 *MET* high polysomy and 13 *MET* gene amplification cases in a cohort of 438 gastric carcinomas. In their study, gene amplification correlated with a poor prognosis.[14] In another study concerning gastric cancer patients, c-MET amplification again correlated and poor survival.[15] In a cohort of 380 non-small cell lung carcinoma cases, Park et al. found that an increase of *MET* gene copy number, present in 11.1% of patients (high polysomy 8.7% and gene amplification 2.4%), was a negative prognostic factor for survival.[13] The relatively low frequency of *MET* mutations and amplification in this OPSCC study cohort could be explained, because *MET* may play a larger role in disease progression in other types of cancer, such as gastric or lung cancer.

Another possible explanation for the low frequency of *MET* mutations and amplification in our study cohort could be that tissue samples were from primary OPSCC's, and not from metastases. There is evidence from previous studies that *MET* mutations are primarily involved in tumor progression to the metastatic phase.[9], [30]–[36] A study by Di Renzo et al. showed that in 4/15 HNSCC cases, activating *MET* mutations undergo clonal expansion during metastatic spread, as their frequency increased from 2% in the primary tumors to 50% in the metastases.[25] *MET* overexpression was also found to be significantly higher in tumor stages associated with enlarged or multiple (N2-N3) lymph node metastases.[37] These findings indicate that the frequency of *MET* molecular abnormalities could be higher in metastatic tissue than in primary tumors, such as the tissue samples studied in our cohort.

Protein expression analysis showed that out of 107 analyzable cases, 46 cases (43%) were c-MET negative, and 61 cases (57%) were c-MET positive at the tumor cell level. We found no significant relationship between *MET* expression and survival. This is in contrast with previous studies showing that *MET* expression is an early event in HNC carcinogenesis[38], and has an association with a poorer overall survival rate[19], [39]. Later, reports describe that compartment localization of c-Met is linked to differentiation and stage of OPSCC tumors.[19] In one study, HNSCC's and their metastases were analyzed, showing that *MET* expression was increased from 2- to 50-fold in about 70% of tumors.[37] Kim et al. previously reported that elevated HGF/c-MET expression in HNSCC correlated with tumor progression[40], and later showed that survival was significantly affected in patients with c-MET expression in SCC of the oral tongue[21], [41].

In previous studies analyzing *MET* expression by IHC, various guidelines were used to classify data. For example, different cut-off values have been used to determine a positive result, such as *MET* expression in >10% of tumor cells[42] or *MET* expression in >30% of tumor cells[43]. These variations in data interpretation can cause the same raw data to yield different (possibly misleading) conclusions.

Unfortunately in our study, analysis of p-MET expression was only possible for select cases, since the AFA fixative caused complete disappearance of p-MET immunostaining. In the future, AFA fixative must be avoided when p-MET IHC is done on archival biopsies. Another factor that may have affected the incidence of *MET* molecular abnormalities was a relatively large number (62%) of HPV positive patients in our sample set. HPV dependant carcinogenesis may partially explain the relatively low incidence of *MET* abnormalities. It is even possible that HPV is a stronger predictive biological marker than *MET.* [4], [44]–[46] Our data does not suggest a relationship between HPV status and *MET* abnormalities.

Our study indicates that *MET* may not be a reliable prognostic biological marker in primary OPSCC, and therefore that anti-*MET* therapy may not be the ideal therapeutic option for these types of tumors. Except for HPV status, we lack reliable diagnostic and prognostic molecular markers for HNSCC, including OSCC. Screening for *MET* molecular abnormalities in primary OPSCC may not be efficient, partly because previous studies have indicated that *MET* abnormalities are predominantly detected in case of metastases. Detection of *MET* abnormalities might be more appropriate as a marker of response to treatment. In a pilot study, Druzgal et al. compared pre-treatment and post-treatment cytokine levels in HNSCC, and found evidence for a strong relationship between HGF serum levels and both therapeutic response and survival.[47] Uchida et al. studied *HGF/MET* in oral SCC and found significantly higher HGF concentrations in metastatic cancer tissues, than non-metastatic or normal tissue. A decline in serum HGF was seen in tumor-free survivors.[30] Dysregulation of the *MET* pathway plays a role in various human cancers, though ‘*MET* addiction’ only occurs in a small percentage of these cancers. There are tumors that only partially depend on *MET* signaling for growth and metastasis, which may be more difficult to detect using biomarkers.[11] Identification of tumors carrying the relevant genetic abnormality and methods for patient stratification according to *HGF/MET* expression requires further investigation to guarantee clinical benefit.[10]

## Conclusions

There was a low frequency of *MET* mutations and amplification in this OPSCC cohort. There was no association between *MET* molecular abnormalities and patient survival. Our results indicate that these *MET* genetic abnormalities may not be reliable prognostic biological markers in OPSCC for patient selection for anti-*MET* therapy.
